# Why the dipolar response in dielectrics and spin-glasses is unavoidably universal

**DOI:** 10.1038/srep29021

**Published:** 2016-07-01

**Authors:** Eduardo Cuervo-Reyes

**Affiliations:** 1 Swiss Federal Laboratories for Materials Science and Technology (Empa), Materials meet life, Dübendorf, 8600, Switzerland

## Abstract

Materials response to electric or magnetic fields is often dominated by the dynamics of dipoles in the system. This is for instance the case of polar dielectrics and many transition metal compounds. An essential and not yet well understood fact is that, despite their structural diversity, dielectric solids exhibit a striking universality of frequency and time responses, sharing many aspects with the behaviour of spin-glasses. In this article I propose a stochastic approach to dipole dynamics within which the “universal frequency response” derives naturally with Debye’s relaxation mechanism as a special case. This formulation reveals constraints to the form of the relaxation functions, which are essential for a consistent representation of the dynamical slowing-down at the spin-glass transition. Relaxation functions with algebraic-, and exponential-tails, as well as damped oscillations, are shown to have a unified representation in which the stable limit of the distribution of waiting-times between dipole flips determines the present type of dynamics.

According to Debye’s model^1^, the polarization developed by a system of dipoles in response to the influence of an external field, relaxes exponentially as a consequence of thermal fluctuations once the field is switched off. However, conclusive evidence collected over many decades indicates that this does not hold true for a wide range of solids^2^,^3^,^4^,^5^,^6^,^7^,^8^,^9^,^10^,^11^,^12^,^13^,^14^,^15^,^16^,^17^,^18^,^19^. A prominent example is the slowing-down of the spin dynamics associated with the on-set of a glassy phase. This problem has received considerable attention over the last decades, yet a coherent description remains elusive. The spin-relaxation function is often modelled^2^,^3^,^4^,^5^ with a stretched-exponential, also known as Kohlrausch-Williams-Watts (KWW) function,


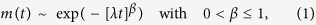


where both parameters, *β* and *λ*, decrease as temperature is lowered. Equation (1) is very appealing since it seems to describe a variety of phenomena such as the conductivity close to the metal-insulator transition^20^, glass forming liquids^21^,^22^, and the jamming transition^23^. However, experimental^5^,^6^ and numerical^7^ evidence indicate that it does not represent adequately systems exhibiting critical behaviour at the freezing temperature, *T*_*f*_. In such cases, the return to equilibrium over long observation times can be modelled more accurately with


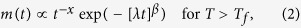



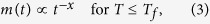


where 0 < *x* < 1, varying from *x* ~ 0.5 around 4*T*_*f*_ to nearly zero well below *T*_*f*_. The emergence of a purely algebraic relaxation (when *λ* → 0 as 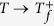) is compatible with the scaling theory of critical phenomena^7^,^24^,^25^, but a microscopic model that leads to such a behaviour is still needed for a deeper understanding. Moreover, the algebraic tail with 0 < *x* < 1 is also a universal feature for the dielectric relaxation of solids^11^,^12^,^13^,^14^,^15^, which may hint at the existence of some underlying generic constraints.

A sound explanation for this ubiquitous behaviour should not rely on very specific assumptions. For instance, models invoking independent parallel relaxation channels are not appropriate as it is very improbable^11^,^12^,^13^,^14^,^26^ that equation (3) results from a superposition of (Debye-)exponentials with a continuous distribution of relaxation times. In addition, no proper (normalized) distribution of exponentials can result in a non-integrable algebraic decay.

More recently it has been proposed^17^,^18^ that a general relaxation equation,


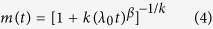


of purely stochastic origin^27^, could also describe spin-glasses across *T*_*f*_, in which case *k*(>0) is related to the non-extensiveness of the system and *k* → 0 above *T*_*f*_, transforming equation (4) in to equation (1).The large-*t* exponent *x* ≡ *β*/*k* goes continuously from *x* ≪ 1 well below *T*_*f*_, to *x* ≫ 1 above *T*_*f*_. However, the corresponding relation between *x* and the relevant time scale known as average relaxation time^7^,


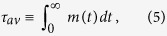


seems to be at odds with scaling arguments^7^,^24^. While *τ*_*av*_ should be finite in the high-*T* phase and diverge at *T*_*f*_, the resulting *τ*_*av*_ from equation (4) does not diverge at the critical point, but at a higher temperature. I.e., the fit to experimental data^18^, and numerical simulations of Ising spin-glasses^7^ give 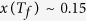, but equation (4) predicts the divergence of *τ*_*av*_ already at *x* = 1 (see Methods). At present, it is neither clear why power-law relaxations are so ubiquitous in nature, nor is there consensus on the description of its transformation across *T*_*f*_.

In this report I propose a stochastic approach to the macroscopic response, which represents the dynamics in both (ergodic and glassy) phases correctly and explains why the response in Ising-like dielectrics and spin-glasses is unavoidably universal. With this formulation we do not aim at solving specific dynamical models and predicting their relaxation exponents. Instead, it allows us to show that the response of any macroscopic system of dipoles must have certain asymptotic forms, and must show relaxation exponent within specific ranges; for instance, 0 < *x* < 1 and 0 < *β* ≤ 1. The global response is reformulated in terms of a distribution of waiting times, whose stable limit determines the type of dynamics. Three types of solutions compatible with fundamental physical principles are derived. One class leads to algebraic decays, another to short-tail relaxations, and the third type corresponds to oscillatory responses. Asymptotic forms, and commonly used interpolation functions are discussed. The present approach supports the evidence^6^,^7^ that above *T*_*f*_ neither equation (1) nor equation (4) describes the magnetic relaxation of a system that undergoes a continuous phase-transition.

## Results and Discussion

### Stochastic formulation of dipole relaxation

Our system consists of a large number of identical spins, or dipoles in general, in equilibrium with a thermal bath. We want to find a generic expression for the time evolution of an induced polarization after the polarizing field has been removed. Evidence indicates that Ising-like models describe well a large number of systems, presumably due to the fact that the rotational SU_2_ symmetry is often broken in solid materials. Therefore, we restrict our analysis in the present work to Ising-like dipoles and assume in the following that each spin can take two values, +1 and −1. This choice also represents any set of two-state variables. Generalisations to other spin values are left for future investigations.

At finite temperatures, each spin can invert its orientation after more or less random time-intervals, being driven by thermal fluctuations, and by the interaction with the other spins in the system. Let us imagine that we follow the dynamic of every spin, writing down the times elapsed between consecutive flips, and that a histogram of waiting-times (in bins of width Δ*t*) is created for each spin. If the system is ergodic, one should find that all histograms become practically identical and reproducible, once the dynamics are recorded for long enough time. The limit Δ*t* → 0 (with a proper normalization) defines the continuous probability distribution function (PDF) of waiting times. For non-ergodic systems, the shape of the histogram may depend on the chosen spin, and may change from one realization of the experiment to another. However, as long as the system size, *L*, is much larger than the spin-spin correlation length, *ξ*, the global PDF obtained by averaging the histograms of all spins, *ψ*(*t*), is reproducible. Although local conditions may be different for each spin due to their interactions, focusing at a global scale in which 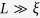 guarantees that there is always a pair of uncorrelated sites in the system where spins have similar conditions with opposite orientations. The dynamics of up- and down-spins are statistically equivalent (in global sense) and their response to small perturbations can be considered linear. In terms of *ψ*(*t*), we can calculate the global likelihood that the number of flips performed by a spin until time *t* is odd,


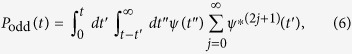


where [*ψ*]^*j^ denotes the *j*^th^ convolution of *ψ*. The average polarization value at time *t*, among the spins that were in the state +1 at *t* = 0, is


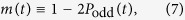


and the same (but with opposite sign) holds for the spins which had the value −1 as initial condition. Thus, equation (7) defines the fundamental solution for the global relaxation function, *m*(*t*) with *m*(0) = 1, and allows us to calculate the global moment at any later time as [*N*_↑_(0) − *N*_↓_(0)] · *m*(*t*) (given the initial number of dipoles up and down, *N*_↑_(0) and *N*_↓_(0), respectively).

A key point in equation (7), which makes it fundamentally different to the stochastic approach leading to equation (4), is that relaxation is not described as the probability that a system remains in its initial state. Instead, it is given here by the (measurable) remaining polarization despite multiple flips. This takes into account that fluctuations are always present, and some transitions do not change the value of the macroscopic observable. To better understand the difference between the two definitions, let us take the extreme case of non-interacting dipoles that invert their orientation periodically. While the probability of not making any transition fades at half of the oscillation period, the spins never forget their initial phase (because the movement is periodic) and the polarization oscillates.

For the simplicity of this formulation based on the global statistics, we pay the price of not knowing about local quantities. In return, the current approach provides a closed-form expression for the relaxation function, without the need for assumptions on the statistical independence of microscopic variables. As a matter of fact, the macroscopic response of spin-glasses and disordered solids is generally reproducible despite the broken ergodicity at microscopic level. In terms of the Laplace transforms, 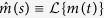 and 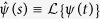, equation (7) acquires a simple form


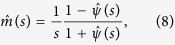


and the frequency-dependent dipolar current is


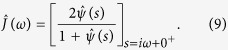


For the time-domain counterpart of equation (9) we have


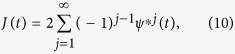


which, by definition (*J*(*t*) ≡ −*dm*/*dt*), fulfils the integral condition 
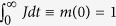
.

The dynamic response of any dipolar system is then expressed, via equation (10), in terms of the corresponding global PDF of waiting times, *ψ*(*t*), which still has to be found by other microscopic methods. But we have not just transferred the problem of calculating *J*(*t*) to the one of finding *ψ*(*t*), which is not necessarily simpler. We have actually gained in that the present formulation unveils important constraints to the possible forms of *J*(*t*), even without further knowledge of *ψ*(*t*). In the remaining of this report we address the following points: (1) the high-frequency universal form of the response is related to the causality principle (this result does not depend on our definition of *m*(*t*)); (2) the stochastic character of the long time response leads to a universal low-frequency form; (3) only few asymptotic long-time forms are physically observable and correspond to different values for the stability exponent of the stable limit on *ψ*(*t*); (4) applying these results to commonly used relaxation functions, we unveil limitations of some specific models and show which of them can describe a continuous phase transition.

### Universality

#### High frequency-short time response

The natural requirement of causality applied to the definition of *J*(*t*) reveals an important constraint to the asymptotic high-frequency form of 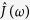. In general, *m*(*t*) is a continuous function with negative derivative in the vicinity of *t* = 0, but it is not necessarily differentiable at *t* = 0. Thus, its power series may have a leading dependence 1 − *m*(*t*) ∝ *t*^1−*n*^, with a non-integer exponent, 1 − *n* > 0, in general. By verifying that 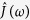 satisfies the Kramer-Kronig relations^28^ (i.e., the causality principle in frequency domain), it is found that *n* must lie within the interval 0 ≤ *n* < 1, and that


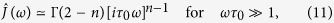


where *τ*_0_ is a time constant, and Γ(⋅) is the gamma function. It turns out that only in the given range of *n* the stored energy and the loss function are non-negative; i.e., 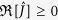 and 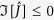 (where 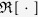 and ℑ[⋅] refer to the real and the imaginary part of a complex function, respectively). For *n* = 0, *m*(*t*) is also differentiable at the origin, and the leading asymptotic behaviour is purely lossy. This is featured in Debye’s relaxation model and corresponds to losses by friction forces that are proportional to the velocity. Although this model provides an easy to understand scenario due to its analogy with the interaction of macroscopic objects with fluids, its accuracy in the (non smooth) molecular world is debatable. For the more general case 0 < *n* < 1, real and imaginary parts of 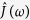 have the same asymptotic dependence, ∝*ω*^*n*−1^, meaning that the polarization-to-losses ratio is frequency independent. This behaviour is actually found in many solids, and liquids^11^,^14^,^29^,^30^, and corresponds to relaxation currents that follow for short times (i.e., for *t* ≪ *τ*_0_) the Curie-von Schweidler law^31^,^32^,


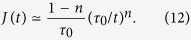


Jonscher^11^, and Dissado & Hill^33^ discussed a simple but brilliant view of dynamical screening at microscopic level, which leads to such a constant loss-tangent over a wide frequency range. Jonscher reasoned that the constancy of the ratio energy-loss/energy-stored in response to a field must be a fundamental dynamical principle. Here we restate the universality of this feature without making reference to any specific dynamical model. It appears simply linked to the causality, and general analytical properties of the response. In addition, Debye’s behaviour, 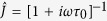, is naturally included as a limiting case.

#### Low frequency-long time response

Let us now turn our attention to the low frequency limit, where the present approach offers a unique insight. From equation (9), taking into account that *ψ*(*t*) (as any PDF) must be non-negative and normalised, one finds that 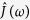 must admit a power series expansion in the vicinity of *ω* = 0; i.e.,


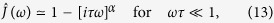


with *α* > 0, where *τ* is another characteristic time. The range of *α* can be inferred from purely stochastic considerations noting that it must be in accordance with the *α*-stable limit of *ψ*(*t*)^34^,^35^. In more detail, *J*(*t*) is dominated at long times by high-order convolutions of *ψ*(*t*), which converge asymptotically to a stable distribution, with 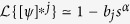 to leading order. The *b*_*j*_s are coefficients which do not depend on *s*. Since *ψ*(*t*) is normalized and non-negative, *α* can only take real values in the interval (0, 2]^36^, which gives from equation (13) a non-negative loss-function, as it should be.

A fundamental consequence of the definition via equation (7) is the asymptotic expression


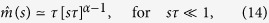


which naturally results in a classification of the dynamics based on the exponent *α*. As 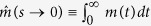, three cases are quickly recognized. For 0 < *α* < 1, the time-integral of the relaxation function diverges. For *α* = 1, 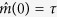, in which case *τ* is equivalent to the average relaxation time, *τ*_*av*_. Last, the integral of *m*(*t*) vanishes for 1 < *α* ≤ 2. As I shall show later on, these three cases correspond to quite distinct relaxation functions. Thus, by defining the macroscopic relaxation function via equations (6) and (7), three different types of dynamics are represented in a unified manner. Equations (11) and (13) with 0 ≤ *n* < 1 and 0 < *α* ≤ 2 restrict the possible asymptotic forms of the response. Time constants, *τ* and *τ*_0_, are system specific and may in general depend on temperature, either directly or through *n* and *α*.

### Glassy systems

Solutions with 0 < *α* < 1 represent glassy systems; i.e., systems which lack of a finite *τ*_*av*_. It follows from equation (14) and the Tauberian theorem, that the relaxation function always has a power-law tail


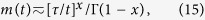


with *x* ≡ *α*. In the limit *x* → 1, Γ(1 − *x*) → ∞ and the algebraic tail disappears, making the way for the short-tail decays that correspond to *α* = 1. It may be worth anticipating that for 1 ≤ *α* < 2, equation (14) does not give power-law decays. Instead, other functional forms are obtained which will be discussed later on. This is in full agreement with considerable amount of data^15^,^14^, where dielectric relaxation with power-law tails is only observed for 0 < *x* ≡ *α* < 1. Furthermore, the algebraic decay in spin-glasses also satisfies this constraint^6^,^7^. From measurements of the relaxation current in many dielectrics^14^ it is known that the log-log plot of *J*(*t*) vs *t* consists of two smoothly connected straight lines, with slopes in the ranges (−1, 0) and (−2, −1) for short and long times, respectively. Those slopes correspond within the present approach to −*n* and −1 − *α*, and therefore, the range of values are naturally constrained. As a consequence of equation (13), 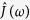 obeys the constant phase relation


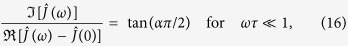


and as shown before if *n* ≠ 0, it also satisfies


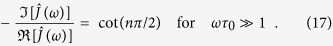


Equations (16) and (17) accurately represent Jonsher’s experimental finding on the ubiquitous constant-phase response of dielectric polymers. Known as Jonscher’s^11^,^14^ universal laws, these relations are considered experimental signatures of a non-Debye relaxation.

#### Dielectric-loss peak

**Figure 1:**
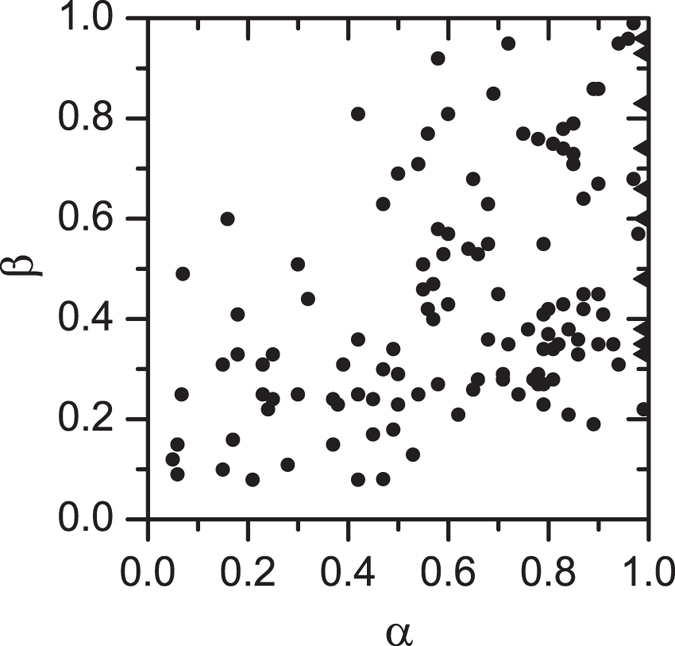
Pairs (*α*; *β*) extracted from the slopes of 130 loss-peaks from the literature^12^,^29^, ^30^. Triangles correspond to pairs with *α* = 1. Loss-peaks are commonly obtained using an impedance spectrometer. Materials considered were both organic and inorganic solids, as well as liquids (see cited refs 12,29,30 for more details).

The presence of loss-peaks is a characteristic feature for the loss-function, 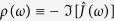, of dipolar systems. In log-log plots of *ρ*(*ω*) vs. *ω*, the regions on either side of the peak maximum are approximately straight lines^12^,^13^. This frequency dependence is determined by the universal relations (11) and (13), giving 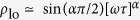 and 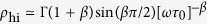 with *β* = 1 − *n*, for 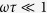 and 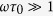, respectively. Although the best form to interpolate between these two limits is still an open question (and multiple models seem to give a good overall fit^12^,^29^,^30^) the common result is that the slope parameters extracted from the experimental data always fall in the predicted ranges, 0 < *α* < 1, and 0 < *β* ≤ 1^11^,^12^,^13^,^14^,^29^,^30^ (shown in Fig. 1).

### Short-tail relaxation

That the time-integral of *m*(*t*) is finite for *α* = 1 is (according to equation (14)) a manifestation of a much stronger condition. The causality principle ensures^28^ that if 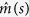 is finite, it is also analytic at the origin. This means that 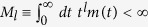 for any *l* ≥ 0, which is only possible if *m*(*t*) exhibits some sort of strong cut-off. (Stretched-)exponentials, and of course also faster than exponential decays fall into this class. Without reference to a specific dynamical model, there is certain freedom for the exact form of *m*(*t*). Nevertheless, all relaxation functions with finite integral have the asymptotic low-frequency response


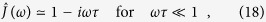


and only in those cases *τ* ≡ *τ*_*av*_ (i.e., the the average relaxation time is finite and is equivalent to the characteristic time-scale *τ*). The analytical relations between 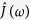 and *m*(*t*) also provide an explanation for equation (2) to fit the data of spin-glasses above *T*_*f*_ better than equation (1) in those cases where the behaviour at *T*_*f*_ is critical^6^,^7^.

#### Describing the critical slowing-down

To describe a continuous phase transition, *m*(*t*) should provide us with a parametric representation of the divergence of *τ*_*av*_. Simultaneously, the parameters *τ*_0_, *τ* and *β* must correspond to a physically meaningful loss-function. Equation (2) satisfies these requirements, and equation (1) does not. Regardless of the form chosen to interpolate between the tail given by equation (2) and the initial condition *m*(0) = 1, the leading behaviour of *τ*_*av*_ as *λ* → 0 is


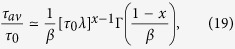


which diverges as *τ*_*av*_ ∝ *λ*^*x*−1^. This does not affect the form of the algebraic factor (*m*(*t*) ∝ *t*^−*x*^) which allows for a physically meaningful frequency response with a short-time scale, *τ*_0_, that remains finite in the limit *λ* → 0. As *τ*_*av*_ diverges, the range of validity of equation (18) shrinks to zero frequency, while equation (18) is being replaced by the form corresponding to the emerging glass phase. This transformation, which starts at the high frequency end (also shown by the decrease of *β*), extends to lower frequencies as *λ* → 0. At *λ* = 0, the low frequency form of 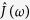 has been completely taken by equation (13) with *α* = *x*, where *τ* is not any more given by equation (19) and it is not the (now divergent) average relaxation time.

For equation (1) on the other hand, *τ*_0_ ≡ *λ*^−1^ and


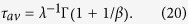


As such, *τ*_*av*_ only diverges if either *λ* or *β* vanishes. However, neither of the two options seems reasonable, because the system would not relax at all (i.e., *m*(*t*) ≡ 1) at finite temperature. While there is no evidence that *β* goes below 0.3 in spin-glass systems^3^,^6^,^7^, the limit 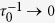 would imply according to equation (11), that the critical system has no losses at finite frequencies. The KWW function may only be adequate for systems which do not have a second order phase transition at finite temperature. This may explain why unusual features were recently found employing equation (1) to fit the spin relaxation data for the solid solution Ba_1−*y*_Eu_*y*_Si; a system that seems to have a true phase transition from a paramagnetic high temperature phase to a glassy one at lower temperatures^5^.

For completeness, let us briefly recall the case of equation (4). As shown before, the type of algebraic decay given by this Ansatz for *k* > *β* corresponds to solutions with *α* < 1 (glassy phase) and has therefore the correct functional form for *T* ≤ *T*_*f*_. However, equation (4) with *k* ≤ *β* is not compatible with our fundamental equation (14). While this *m*(*t*) has a finite time-integral for *x* ≡ *β*/*k* > 1, all moments 
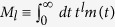
 of order *l* ≥ *x* − 1 diverge, in contradiction with the requirement of analyticity for 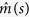. This indicates that equation (4) cannot represent a causal response in the high-temperature phase and that equation (1) cannot be considered (in the present physical context) as the *k* → 0 limit of equation (4), in contrast with previous thoughts^17^,^18^,^27^. This conclusion does not depend on any particular definition of the average relaxation time. It is a direct consequence of the analytical properties imposed on *m*(*t*) by the causality principle (and the equivalent Kramers-Kronig relations for 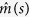)^28^. i.e.; if *m*(*t*) has finite integral, then all the higher moments must be finite. It should be mentioned that the discrepancy between equation (4) for *k* ≤ *β* and equation (14) does not necessarily imply an inconsistency of the model leading to equation (4). The latter represents the probability that the system remains in its initial microscopic state, which should not be compared directly with the data of macroscopic relaxation.

### Constraints on specific models: Assessments made simpler

Equations (11), (13) and (14), and their time-domain counterparts have been derived here without reference to any dynamical model and as such should be satisfied by any macroscopic Ising-like dipolar system. Although these equations only restrict the asymptotic forms, they can be employed for checking the consistency of models used to fit experimental data. This is a useful and quite unique tool as the true asymptotic behaviour of a Fourier transform is often difficult to asses with purely numerical evaluations.

Let us consider, for instance, the KWW decay. Following its extensive use for analysing relaxation data, great effort has been also put into finding an accurate representation of the corresponding 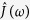
37,38,39,40,41, of which there is no exact analytical form. Particularly, since the frequency response of dielectrics is well represented by the Havriliak-Negami (HN) equation^30^,


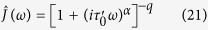


there have been multiple intents to prove its equivalence with the KWW decay and to find the mapping between their form-parameters. Earlier results indicated that not for all values of *α* and *q* equation (21) would Fourier-transform into something similar to the time response, *J*(*t*) = −*dm*/*dt*, obtained from equation (1)^37^. More recently, it was proven that both functions are always distinguishable and values of *α* for the best fits were also reported, taking apparently the whole range 0 < *α* ≤ 1 as a function of the KWW-parameter *β*^39^. However, from a recent method using asymptotic series expansions^40^ it can be deduced that the KWW function should always correspond to *α* = 1, in contrast with the previous numerical results in the literature^37^,^39^.

The present approach brings a simple solution to the problem of equivalence between the KWW and the HN functions. A straightforward result from equation (14) is that, indeed, the correct asymptotic behaviour requires *α* ≡ 1, in agreement with the method from ref. 40. The HN equation has a low-frequency expansion 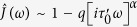 which can only correspond to a KWW *m*(*t*) (with finite *τ*_*av*_) for *α* = 1. We can check for the equivalence a bit further. Generally, the misfit between two functional forms may be hidden with an independent adjustment of several form-parameter, which could lead to incorrect dependences between some of these variables. An advantage of knowing the asymptotic forms is that we can fix several of the form-parameters and therefore reduce the (misleading) degrees of freedom. In the present case requiring full consistency between the asymptotic forms of the KWW and the HN functions, one finds that *β* = *qα*, 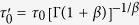 and


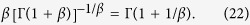


**Figure 2:**
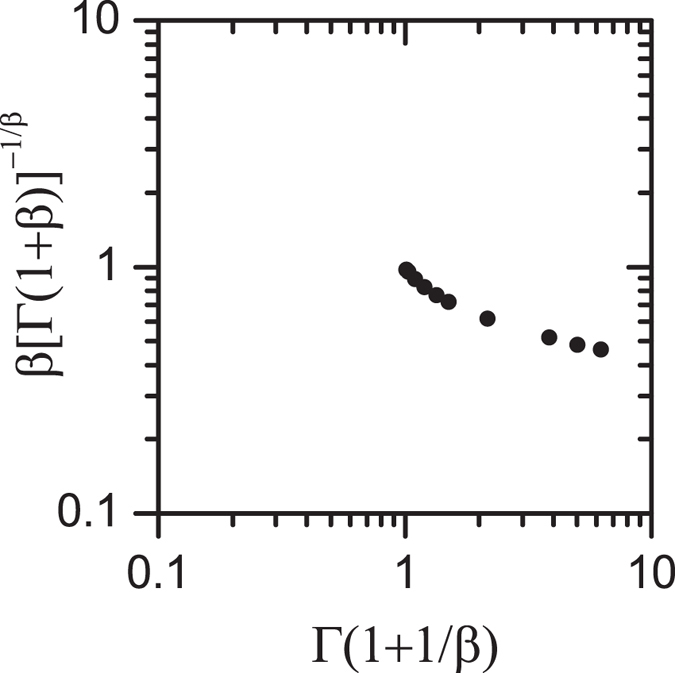
Test showing the general non-equivalence of the KWW decay and the HN response. Equivalence requires that the data points fall along the diagonal (see description in the text). The points correspond to *β* values form actual materials^12^,^29^,^30^.

These equalities must be simultaneously satisfied. The latter represents the equivalence of the ratio *τ*/*τ*_0_ obtained from the analytical form of the KWW decay (r.h.s) and that obtained from the HN response (l.h.s.). Only if both sides of equation (22) are similar within certain error margin for all *β*, one can then say that KWW and HN functions are equivalent. We actually find that they are not equivalent, as it can be seen from the off-diagonal distribution of the data points in Fig. 2 evaluated for the *β* values of actual materials^12^,^29^,^30^. The knowledge of the asymptotic relations makes the assessment of equivalence simpler, and free of numerical errors.

A similar test can be done comparing the HN response with the relaxation given by equation (4). The consistency of the asymptotic forms in real-time requires that the parameters in equation (4) satisfy 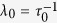, *β* = 1 − *n*, *k* = (1 − *n*)/*α*, and


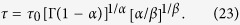


**Figure 3:**
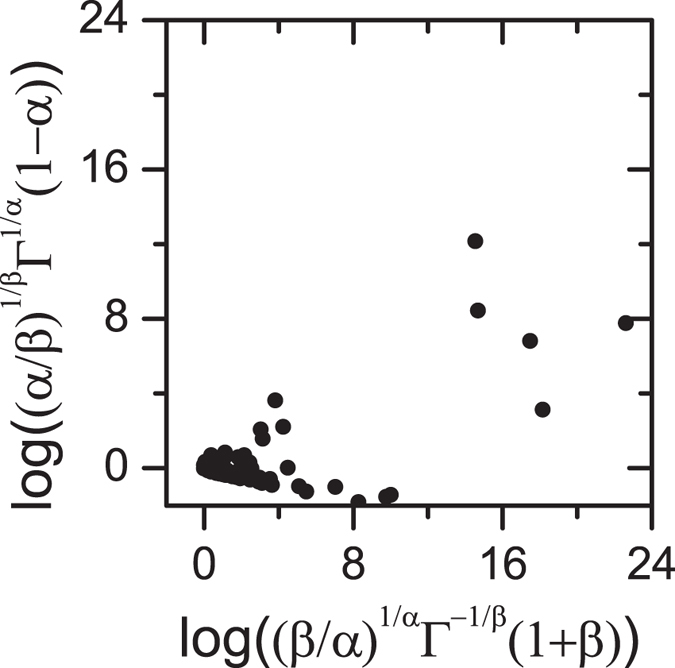
Test showing the general non-equivalence of the HN response and equation (4). Equivalence requires that the data points fall along the diagonal (see description in the text). The points correspond to (*α*; *β*) values form actual materials^12^,^29^,^30^.

From the HN equation one obtains as before


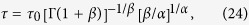


and requiring the equivalence of the two representations leads to


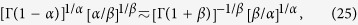


for any (*α*; *β*). The l.h.s. vs. r.h.s. plot of equation (25) is shown in Fig. 3 for about 100 dielectric materials (liquids, and solids), taking the (*α*; *β*) values reported in the literature^12^,^29^,^30^. The strong departure from the diagonal indicates the non-equivalence of the time response given by equation (4) with the frequency response from equation (21).

To date, a relaxation model which gives a coherent representation of the HN response in the general case is not yet known. From a different perspective, the HN function is not the only existing parametrization of the dipolar loss^12^,^29^,^30^ and it is not clear whether it always gives the most complete description. The answers to these questions might be found with the help of the asymptotic relations here presented, but this lies beyond the scope of the present report.

### Other solutions

Definition (7) does not restrict *m*(*t*) to positive-definite functions. For instance, *m*(*t*) = exp(−[*t*/*τ*_0_]^*β*^) cos(*ω*_0_*t*) is also a “short-tail” function that has a finite time-integral and satisfies the natural conditions of equations (11) and (13). Thus, exponentially damped oscillations can also be represented via equation (7).

The third and last class of relaxation functions, corresponding to 1 < *α* ≤ 2, represents a behaviour that is fundamentally different to those previously described. The asymptotic form of 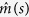 given in equation (14) can be interpreted as the product of two terms; [*sτ*]^*α*−2^ and *sτ*^2^. The first one, [*sτ*]^*α*−2^ with 1 < *α* < 2, is equivalent to the already discussed [*sτ*]^*α*−1^ with 0 < *α* < 1. The second term corresponds to the lowest order expansion of the Laplace transform for the cosine function; i.e., 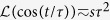 for *sτ* ≪ 1. Thus, the long time behaviour of *m*(*t*) in this case is given by the convolution of an oscillating function with a power-law, which may relate to fluctuations in systems with long-range order. As such, the new formulation of dipolar relaxation given by equation (7) in terms of a global PDF of waiting times, not only allows to explains the universality of the algebraic decay in Ising-like dielectrics and spin-glasses, but also represents several types of dynamics in a unified manner. A key aspect in this approach is that it focuses on the macroscopic response which is reproducible and allows us to deal with non-ergodic regimes as well as with ergodic ones. The asymptotic relations here presented may be used as guidelines for the creation of consistent relaxation models and the analysis of experimental data. Detailed calculations and further discussion about the relation of this approach with other microscopic models will be presented elsewhere. The possibility of a generalisation to higher spins (more than two-state systems) should be addressed in future works.

## Methods

### Definition of the average relaxation time

The response function *J*(*t*) relates the induced polarization *p*(*t*) to the applied field *E*(*t*)


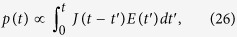


giving how the intensity of the response varies with the time (*t* − *t*′) between input signal at time *t*′ and the measured polarization at time *t*. By definition (*J*(*t*) ≡ −*dm*/*dt*), *J*(*t*) satisfies


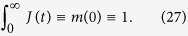


Therefore, it is also interpreted as a probability distribution from which the average response time (or average relaxation time) is calculated as


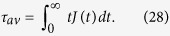


If and only if *τ*_*av*_ exists (when it is finite), the integral in equation (28) can be done by parts, giving 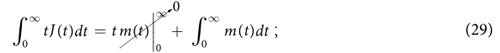


where the integral in the r.h.s. is the equivalent definition presented in equation (5).

### Calculation of the average relaxation time corresponding to equation (4)

For equation (4),


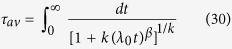


and it is only finite for *x* ≡ *β*/*k* > 1 because of the large-*t* asymptotic behaviour, *m*(*t*)~*t*^−*x*^. Equation (30) gives


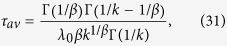


which diverges as


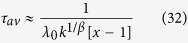


when *x* → 1^+^. Γ(⋅) is the gamma function.

## Additional Information

**How to cite this article**: Cuervo-Reyes, E. Why the dipolar response in dielectrics and spin-glasses is unavoidably universal. *Sci. Rep.* **6**, 29021; doi: 10.1038/srep29021 (2016).
